# Long-Term Outcome of Metal-on-Metal Total Hip Arthroplasty with Modular Neck Stem

**DOI:** 10.3390/jcm13061525

**Published:** 2024-03-07

**Authors:** Hiroki Wakabayashi, Masahiro Hasegawa, Yohei Naito, Shine Tone, Akihiro Sudo

**Affiliations:** Department of Orthopaedic Surgery, Mie University Graduate School of Medicine, Tsu 514-8507, Japan

**Keywords:** long-term outcome, metal-on-metal, modular neck stem, total hip arthroplasty

## Abstract

**Background**: This study aimed to report the long-term outcomes of total hip arthroplasty (THA) using a Conserve Plus (Wright Medical, Japan) metal-on-metal (MoM) acetabular prosthesis with a modular neck stem. **Methods**: This study enrolled 50 patients (10 men and 40 women; mean age, 65.8 (39–87) years) who underwent primary THA using a Conserve Plus MoM acetabular prosthesis with a modular neck stem. The preoperative diagnosis in most patients was osteoarthritis. Clinical function of hip joint outcomes was investigated using the Japanese Orthopedic Association (JOA) hip score preoperatively and at the final follow-up. The perfect JOA hip score was 100, while the worst score was 0. Radiological analyses were evaluated during the final follow-up visit. Magnetic resonance imaging (MRI) images were evaluated to screen for pseudotumors in 43 hips postoperatively. **Results**: Six patients did not visit before their 10-year follow-up for unknown reasons. Therefore, 44 patients were evaluated at a mean of 11-years of follow-up (10–12 years). The mean (±SD) preoperative JOA hip score of 44.2 (±15.5) improved significantly to 85.1 (±12.9) postoperatively at the final follow-up (*n* = 36 hips, excluding eight revision cases). One patient underwent femoral fixation for a periprosthetic fracture due to trauma that occurred 4 years postoperatively. Spot welds were identified in 93.2% (41/44 hips) of cases. Severe (third- and fourth-degree) stress shielding was identified in 40.9% (18/44 hips) of cases. Twenty-two patients (51.2%) had pseudotumors attributable to MoM articulation based on MRI results, 2 to 10 years after arthroplasty. Three hips showed cup osteolysis (7%) and three showed trochanteric region osteolysis (7%). There were seven cup and/or three stem revisions for aseptic loosening and/or osteolysis at 4 months (with trauma) and 3.3 to 11 years (with pseudotumor) postoperatively. The Kaplan–Meier survivorship for the THA construct in this group was constant at 93.0% and 75.9% at 10 and 12 years after arthroplasty, respectively. The rates of survivorship of revision and loss of follow-up at 10 and 12 years were 83.9% and 66.8%, respectively. **Conclusions**: In summary, we reported on the long-term treatment results of MoM THA, precautions based on our cohort’s findings, and the measures taken to address these issues, such as revision replacement and its outcomes. Clinical scores revealed good outcomes during the mean 11-year follow-up period. However, the prevalence of pseudotumors (PTs) was 51.2%. Some cases required revisions even after the 10 years following surgery. This is because in MoM THA, PT occurrence increases over time, and as a result, there were cases in which revised THA was required even after 10 years.

## 1. Introduction

Total hip arthroplasty (THA) is one of the most successful surgical operations and improvement in quality of life [1].

Metal-on-polyethylene implants offer a safe, predictable, and hip-bearing surface for most patients, but they release polyethylene (PE) debris, leading to cytokine and proteolytic enzyme production [2]. PE debris, the main cause of THA failure, induces periprosthetic osteolysis, culminating in aseptic loosening [2].

Metal-on-metal (MoM) bearings were reintroduced in THAs in the 1990s to address polyethylene-related issues, such as osteolysis and loosening from wear, and high rates of dislocation. Large-diameter metal femoral heads increase the range of motion and reduce femoral neck impingement on the acetabular rim, showing promising early results [3,4,5].

Despite its potential advantages, MoM hip arthroplasty, including total hip replacement and hip resurfacing, faces high implant failure rates [6,7]. Hip arthroplasty registries have reported high revision rates, particularly for stemmed MoM hip arthroplasties [8,9].

A continuing concern with MoM articulation is the local and systemic release of metal ion debris in the blood and urine of patients. These metal–metal wear-related complications include the formation of pseudotumors (PTs), metallosis, and soft tissue necrosis—adverse reactions to metal debris (ARMD).

During the past decade, ARMD has been widely studied in patients who underwent MoM hip arthroplasty [10,11].

Soft tissue reactions, termed PTs [10], are increasingly identified in MoM hips, with terms such as ARMD [11], adverse local tissue reactions, and aseptic lymphocyte-dominated vasculitis-associated lesions [12] used as umbrella terms for these lesions.

However, studies on the long-term results of patients undergoing MoM THA, particularly concerning ARMD and revision rates, are limited. Even if MoM-bearing surfaces are no longer used, long-term data could help define the course and best management for these patients. In our recent report involving patients with modular Pinnacle MoM THA (DePuy), screening with MRI confirmed a 29% incidence of ARMD in patients [13].

Radiography and MRI can determine the extent of tissue destruction, with larger cup abduction angles (acetabular inclination) previously associated with an increased risk of failure [11,13]. It has also been surmised that the actual source of metal debris in these MoM THAs may be the trunnion junctions [11,14].

This study reports long-term clinical outcomes and radiological findings, including magnetic resonance imaging (MRI) results for THA with Conserve Plus (Wright Medical, Japan) MoM acetabular prosthesis with a modular neck stem.

## 2. Materials and Methods

This study enrolled 50 patients (10 men and 40 women; mean age, 65.8 (39–87) years) who underwent primary THA using a Conserve Plus MoM acetabular prosthesis and Profemur TL modular neck stem (Wright Medical, Japan), with most patients having a preoperative diagnosis of osteoarthritis.

The shell, a high-carbon-cast cobalt–chrome acetabular component with a porous/HA-beaded surface, facilitated biological fixation. A low-profile hemispherical shell allowed 170° of articular coverage.

The femoral stem, uncemented with a flat tapered wedge, featured a modular titanium alloy femoral neck providing 12 possible combinations of neck-stem angle (varus, neutral, and valgus), neck version (anteroverted or retroverted), and neck length (short or long).

### 2.1. Clinical Evaluation

Clinical function of the hip joint was investigated using the JOA hip scores preoperatively and at the final follow-up visit. The JOA hip score consists of the following four subcategories: pain (up to 40 points), range of motion (ROM; 20 points), walking ability (20 points), and activities of daily living (ADL; 20 points). The perfect JOA hip score was 100, while the worst score was 0 [13].

### 2.2. Radiological Evaluation

Radiological analyses were performed during the final follow-up. Anteroposterior (AP) and lateral radiographs of hip joints were obtained for each patient and analyzed by an experienced radiologist and an orthopedic surgeon. Osteolysis was determined as radiological analysis using the Gruen and DeLee classifications [15,16]. Stress shielding, reactive lines, spot welds, and sclerosing were examined using radiological analysis according to Engh [17]. In addition, the inclination angles of the acetabular components were measured [18]. Femoral offset (FO) was measured as the distance from the center of rotation of the femoral head to a line dissecting the long axis of the femur [19]. Additionally, the ratio of the FO (RFO) on the operated hip to that on the contralateral hip was calculated.

MRI was used for screening of pseudotumors and was conducted in 43 hips postoperatively, excluding one hip with a cup revision for aseptic cup loosening at 4 months after a falling trauma. MRI was first performed 2 years postoperatively and thereafter every 2–3 years until 10 years postoperatively. The incidence of PTs over time was evaluated with MRI following MoM THA.

Primary THA and 50 primary THA procedures (*n* = 50) were performed at a single institution between 2010 and 2011. Forty-four THAs (44 patients) were evaluated at a minimum 10-year and a mean 11-year follow-up (10–12 years). Three patients who died of malignancy and pneumonia at 4, 6, and 9.5 years after surgery, were included.

### 2.3. Survivorship

A revision was defined as the removal of any THA component for any reason, and THA survivorship was defined as the absence of revision. The Kaplan–Meier method was used to estimate THA survivorship, where the time variable for a subject was the time to revision if the THA had been revised, the time to last follow-up, or death if the THA had not been revised.

Institutional Review Board approval was obtained, and the patients provided informed consent prior to inclusion in the study. All procedures were performed in accordance with the ethical standards of the Institutional Committee and the 1964 Declaration of Helsinki and its later amendments.

### 2.4. Statistical Analysis

Statistical analyses were performed using the non-parametric Wilcoxon signed-rank test, analysis of variance, and Spearman’s rank correlation coefficient. Statistical significance was set at *p* < 0.05. Kaplan–Meier survivorship analysis was performed using re-vision for any reason as the endpoint. All statistical analyses were performed using the IBM SPSS Statistics 26 (IBM Japan, Tokyo, Japan).

## 3. Results

**Figure 1:**
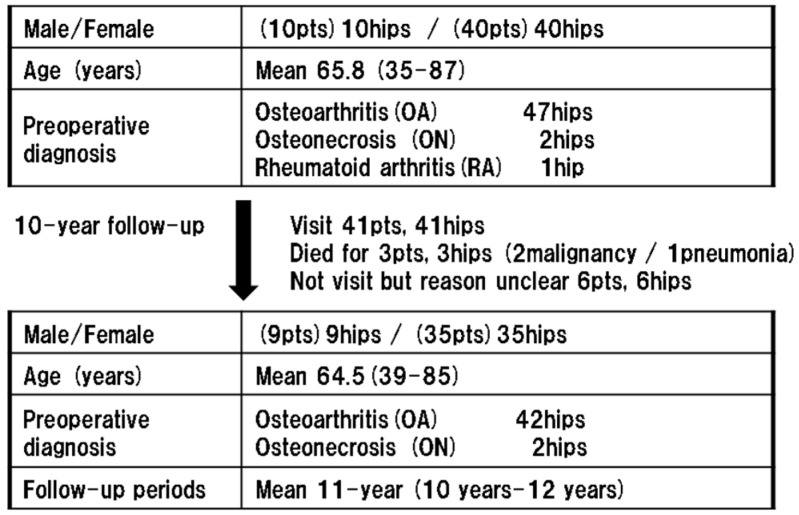
Study flow chart of patient selection and demographic background.

Nine patients (nine hips) were lost before their 10-year follow-up. One patient (one hip) died of pneumonia after 6 years. Two patients (two hips) died of cancer after 4 and 9.5 years, respectively. However, a final follow-up was performed on three patients. Therefore, these patients were included in the present study. Another six patients (six hips) did not visit before their 10-year follow-up for unclear reasons. After excluding the six patients who were lost to follow-up, 44 patients (44 hips; 9 men and 35 women; mean age, 64.5 (39–85) years) were evaluated at a mean 11-year follow-up (10–12 years). Patient demographics are shown in Figure 1. Primary THA was indicated for primary hip osteoarthritis (OA) in 95.5% (42/44) of patients. The other two patients were diagnosed with avascular necrosis of the femoral head.

One patient underwent femoral fixation for a periprosthetic fracture due to trauma at 4 years postoperatively. No hip dislocations or infections were observed. Forty-three hips were subjected to MRI, excluding one hip for cup revision due to the aseptic cup loosening at 4 months after trauma of falling. The first MRI (2–3 years after surgery) revealed 14 hips (32.6%) with PTs. After that, the number hips with PTs gradually increased (Supplementary Figure S1). Twenty-two hips (51.2%) contained PTs attributable to the MoM articulation as revealed with MRI 2 to 10 years postoperatively with a mean of 4.2 (±standard deviation [SD], 2.5) years. A total of 7 hips (16.3%) were symptomatic and 15 hips (34.9%) hips were asymptomatic. 

In radiological analyses conducted at the final follow-up, spot welds were identified in 93.2% (41/44 hips) of cases. Severe (third- and fourth-degree) stress shielding was identified in 40.9% (18/44 hips) of cases. Osteolysis occurred in three (7%) hips in the acetabulum and 3 (7%) in the proximal femur. Eight hips (eight patients) were switched to metal-on-polyethylene articulation between 4 months and 11 years postoperatively owing to pain, swelling, implant failure, or both (Table 1). PTs were identified in seven hips with ARMD, excluding one hip with a cup loosening 4 months after the trauma of falling (Case 1). The mean duration for the observed PTs after primary arthroplasty was shorter for patients with revision surgery for ARMD (3.9 years) compared with those who had PTs but did not receive revision surgery (4.3 years), but the difference is not significant. MRI images revealed PTs in some revised cases and these can be seen in Supplementary Figure S2. Of the seven revised cups, six exhibited cup loosening (Case 1–6), one cup exhibited osteolysis (Case 7), and of the three revised stems, one exhibited osteolysis (Case 5) and two exhibited periprosthetic femoral fractures (Cases 4 and 8; one occurred during surgery) (Supplementary Figure S3). Some cases (Case 6–8) required revision even 10 years after THA (Table 1). In all the revised cases, except for Cases 1 (due to trauma and early revision) and 2 (due to unknown results of surgery due to revisions being conducted at another hospital), had head–neck junction corrosion (six hips), but stem–neck modular junction corrosion was only present in three hips. The hips with well-fixed and well-positioned implants may be treated with an exchange of modular components and limited revision. Our revision cases, except Cases 4 and 5, were treated with an exchange of modular components and limited revision.

**Figure 2:**
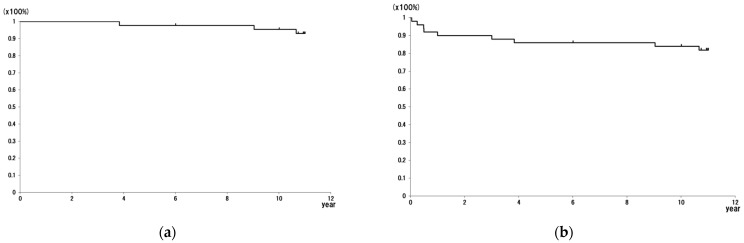
Implant and follow-up survival. (**a**) Survival of revision endpoint. (**b**) Survival of revision and loss of follow-up endpoint. The overall implant survival rates at 10 and 12 years were 93.0% and 75.9%, respectively. The rates of survivorship of revision and loss of follow-up at 10 and 12 years were 83.9% and 66.8%, respectively.

The overall implant survival rates at 10 and 12 years were 93.0% and 75.9%, respectively (Figure 2a). The rates of survivorship of revision and loss of follow-up at 10 and 12 years were 83.9% and 66.8%, respectively (Figure 2b).

We assessed the radiological outcomes in 43 patients (43 hips), excluding one hip that was revised for cup loosening with trauma (falling) 4 months after primary arthroplasty. The mean acetabular cup inclination angle was 45.8° (SD, ±8.8°). The mean anteversion was 11.9° (±4.1°), and the mean RFO on the operated hip to the contralateral hip was 1.0 (±0.11). Stress shielding progressed to grades 3 or 4 in 18 hips (40.2%) during the study period.

Twenty-one hips had no PTs, and 15 had PTs but no revision surgery. Eight patients (eight hips) underwent revision surgery for ARMD and aseptic loosening with trauma. Sex, age, and body mass index (BMI) were not significantly different among the three groups. In the radiological analysis, the mean cup angles of inclination and cup failure (osteolysis, loosening, or both) were the highest in patients with revision surgery for ARMD (54.6°), significantly higher compared with those who underwent PTs but not revision surgery (43.2°) and those who did not undergo PTs (43.8°) (Table 2). The mean RFO and mean cup angle of anteversion were not significantly different among the three groups.

Stem osteolysis tended to be higher in patients who underwent revision surgery compared with those with PTs but no revision surgery and with those who did not have PTs.

**Figure 3:**
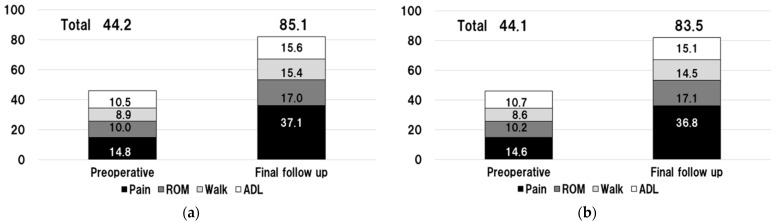
Clinical hip function outcomes using the Japanese Orthopedic Association (JOA) hip score preoperatively and at the final follow-up. (**a**) In 36 patients (36 hips), 8 hip revisions were excluded due to infection after primary arthroplasty. (**b**) In 44 patients (44 hips), 8 hip revisions were performed.

The mean (±SD) preoperative JOA hip score improved significantly to 85.1 (±12.9) postoperatively at the final follow-up (*n* = 36 hips, excluding eight revision cases) (Figure 3a) and to 83.5 (±13.2) when including all patients (*n* = 44 hips) (Figure 3b). No statistically significant differences in JOA hip scores (mean ± SD) were observed between patients without PTs (85.1 ± 14.1) and those with PTs but no revision (85.1 ± 11.2). In patients treated with revision THA, there were significantly improved JOA scores and subcategories of pain and range of motion (ROM) at final evaluation compared to before the revision (Table 3). However, there were significantly lower JOA scores and subcategories of walking and ADL in the final evaluation of patients treated with revision THA than in those without PTs and those with PTs but no revision THA (Table 4).

## 4. Discussion

THA is the treatment of choice for hip disability [1]. Around the turn of the 21st century, there was a re-emergence of metal-on-metal (MoM) total hip implants with the hope to obtain an implant that will have improved survivorship because of the lack of wear created from traditional polyethylene bearings [20]. MoM bearings offer the advantages of lower dislocation and wear rates. Large-diameter MoM heads (LDHs, ≥36 mm) have been shown to increase implant stability and decrease dislocation risk.

However, MoM hip arthroplasty is a poorly studied topic. Approximately 10 years after the peak of its use, MoM hip implant usage decreased dramatically as numerous revisions became apparent. Concerns regarding increased blood metal ions, metallosis, pseudotumor formation, and early failure rates have led to the bearings’ diminishing use [21]. After a promising start, several studies with these second-generation MoM bearings, including LDHs, have shown an increased risk of ARMD and higher-than-expected revision rates [22,23]. ARMD became more widely documented and known and eventually became an umbrella term to encompass a spectrum of reactions involving metal debris including acute lymphocytic vasculitis and pseudotumors [11].

According to the 19th report of National Joint Registry of the United Kingdom (NJR), the most common reasons for revision among THAs are aseptic loosening, dislocation, and ARMD, while MoM bearings have the highest incidence of ARMD [9]. In previous studies with LDH MoM implants, 31–69% of all revisions were performed due to ARMD [24,25], and the prevalence of definite ARMD was 11–14% within a mean follow-up period of 3.8–6.7 years [24,26,27]. In a recent report of long-term outcomes in MoM hip arthroplasty, the 15-year implant survival rate was 69% (confidence interval [CI] 67–71%) for the whole study group, 56% (CI 53–60%) for stemmed primary MoM THAs, and 84% (CI 82–87%) for resurfacings. The 12-year survival for MoM THAs implanted in revision surgery was 66% (CI 59–73%). The most common reason for revision was ARMD both among primary MoM THAs (83%) and hip resurfacings (70%) [28]. Additionally, previous studies have reported a 7%–61% incidence of asymptomatic PTs among MoM hips [29,30,31].

In our previous report, Pinnacle/S-ROM (DePuy) with a MoM bearing was analyzed; at 13 years postoperatively, the survival rate of revision endpoints was 90.4%, and screening with MRI confirmed a 29.0% incidence of ARMD patients (8.7% of symptomatic and 20.3% of asymptomatic patients) [13].

In this study, the first MRI conducted 2 years after primary surgery revealed 14 hips (32.6%) with PTs, and the number hips of observed PTs gradually increased. At 10 years after primary surgery, PTs were found in 22/43 (51.2%) screened hips, and of these PTs, 7/43 (16.3%) hips were symptomatic, and 15/43 (34.9%) hips were asymptomatic. All seven hips that were revised due to PTs experienced symptoms, such as chronic hip pain (Table 1).

The risk factors for component revision were younger age at surgery, higher cup inclination angle, and the female sex [32]. Revisions for ARMD can be complex with extensive bone loss, and damages to the surrounding soft tissue are encountered intraoperatively and are prone to complications and re-revisions [33,34,35]. Three published studies state that suboptimal acetabular cup inclination led to a higher incidence of ARMD and revision rate [11,36,37]. Increased cup angle of inclination was a risk factor for revision in a previous study, with an increased risk of 3% for each increase of one degree [22]. 

In our previous report on Pinnacle/S-ROM (DePuy), the mean cup angle of inclination and mean ratio of femoral offset on the operated hip to the contralateral hip was highest in patients who underwent revision surgery [13]. Radiological analysis revealed that the mean cup angle of inclination was highest in patients who underwent revision surgery for ARMD compared to patients with PTs but no revision surgery and those with no PTs. 

In this analysis, the mean cup angles of inclination and cup failure (osteolysis, loosening, or both) were significantly higher in patients with revision surgery for ARMD compared with those who underwent PTs but not revision surgery and those who did not undergo PTs (Table 2). However, the mean RFO was not significantly different among the three groups. One of the reasons why there was no difference in mean RFO among the three groups may be that the THA with modular neck femoral stems was able to be adjusted to an appropriate offset.

THA with modular neck femoral stems (MNFS) has surged in popularity because it offers the possibility of restoring the patient’s anatomy and further improving stability [38,39,40]. Neck–stem modularity of the femoral component was introduced to allow independent control of not only length and offset but also version. However, modular components with their additional interfaces may be sources of metal ions and metal particles released into the body, potentially leading to an increased rate of ARMD. 

Neck–stem junction corrosion is not completely understood despite the identification of contributing factors having been identified. A titanium stem with a cobalt–chromium head can increase the risk of galvanic corrosion at the taper junction, which is commonly referred to as trunnionosis, particularly when using large heads (≥36 mm) [41]. Known cofactors for this process include a longer neck, varus offset neck, dissimilar metal combination, and intraoperative contamination of the taper interfaces [42].

Several studies have compared MNFS to monoblock femoral stems in THA. In the prospective study comparing patients with the modular neck femoral stem (MNFS) with a matched cohort of patients with nonmodular femoral stem (NFS), Mikkelsen RT et al. [43] found that more patients in the NFS group had pain than the MNFS group. The metal ion levels were higher in the MNFS group, but there was no difference in the presence of PTs between the two groups. More patients in the NFS group had pain than in the MNFS group. It could be that the patients’ anatomy might not be as well reconstructed in the NFS group. However, recent studies have discovered no clinical advantages of MNFS over monoblock femoral stems [43,44]. Duwelius et al. found no difference in leg length, Harris hip score, or short-form 12-item scores between MNFS and NFS THAs. This study investigated MNFS using Zimmer^®^ M/L Taper Kinectiv (Warsaw, IN, USA), which is of modular design with a cobalt–chromium–titanium head–neck junction and a titanium–titanium neck–stem junction [44].

Vendittoli et al. [45] compared ion levels and the rate of ARMD between two groups of 45 patients who underwent unilateral primary LDH MoM THA with the same MoM bearing, 32 patients with monoblock stems and 13 with modular stems. ARMD rate was significantly higher in the modular group (46%) compared with the monoblock group (16%, *p* = 0.031). Revision for ARMD was performed at 52.8 ± 8.1 months in the modular group versus 98.2 ± 15.5 months after primary THA in the monoblock group. ARMD originated from wear and corrosion of the junction between the stem and femoral head adapter sleeve in all monoblock cases, and the junction between the stem and modular neck in all the modular ones. Cr and Co ion levels were significantly higher in the modular stem group (*p* < 0.001 for both). Interestingly, all cases of ARMD in the monoblock stem group originated from wear and corrosion of the junction between the stem and the femoral head adapter sleeve, and all cases of ARMD in the modular group originated from wear and corrosion of the junction between the stem and the modular neck. The problematic junctions for each group included dissimilar metal combinations (Ti connected to Cr–Co). However, this study was not described in radiological evaluation. MoM bearing contact is increased in implant malposition thereby causing edge loading and generating a multitude of metal ions released into the serum and synovium.

Recent studies have shown that ARMD can be observed in THRs composed of a cobalt–chrome (CoCr) modular neck coupled with a titanium alloy femoral stem and a ceramic-on-ceramic bearing. ARMD has been reported mostly in MoM implants and especially in THR with Large Diameter Heads (LDHs). ARMD in these non-MoM implants is caused by fretting at the interface between the neck and stem of dissimilar metals, rather than tribo-corrosion between bearing surfaces [46].

A previous study compared the effects of two bearing types (MoM and ceramic-on-ceramic [CoC]) on the stem–neck modular junction performance of MNFS THA. MoM LDH MNFS THAs exhibited catastrophic stem–neck modular junction corrosion, which resulted in significantly higher Cr and Co ion release and revision rates than their CoC equivalents [47].

The combined Interaction of the LDH MoM bearing and titanium alloy MNFS with the Cr–Co neck proved to be catastrophic and significantly increased metal ion release, related ARMD, and revision surgeries [47].

In our study, the revised cases, except for Cases 1 (due to trauma and early revision) and 2 (due to unknown findings of surgery due to revision at another hospital), had head–neck junction corrosion in all cases (six hips), and stem–neck modular junction corrosion was observed in three hips. The modular neck stem of the Profemur TL has a cobalt–chromium–titanium head–neck junction and a titanium–titanium neck–stem junction. Taper corrosion may represent an additional source of metal debris. The frequency of ARMD was higher and the survival rate of the implant was lower in this study than in our previous report on Pinnacle/S-ROM. We considered that corrosion appeared at the head–neck junction.

When considering revision MoM THA, it is important to realize this is a heterogenic population and not all revision reconstructions are the same. Patients with well-fixed and well-positioned implants may be treated with an exchange of modular components and limited revision [48], whereas malpositioned implants and large soft tissue defects require more extensive revision reconstruction [49]. Our revision cases, except Cases 4 and 5, were treated with an exchange of modular components and limited revision, and Cases 4 and 5 required full revision reconstruction.

The limitations of this study are those inherent to any retrospective study. This study also has a limited sample size, the absence of a control group, a high dropout rate, and the lack of blood Co and Cr data.

## 5. Conclusions

In summary, we reported on the long-term treatment results of MoM THAs, precautions based on our cohort’s findings, and the measures taken to address these issues, such as revision replacement and its outcomes. Clinical scores revealed good outcomes during the mean 11-year follow-up period. However, the prevalence of pseudotumors (PT) was 51.2%. Some cases required revisions even after the 10 years following surgery. This is because, in MoM THAs, PT occurrence increases over time, and as a result, there were cases in which revised THA was required even after 10 years. The current cohort of patients will continue to be followed up, with special attention paid to any potential local tissue reactions or other complications resulting from metal wear debris. This report is expected to serve as an educational guide for patients who have undergone MoM THA and their physicians.

## Figures and Tables

**Table 1:** Patient demographics in revision cases. Seven hips were switched to metal-on-polyethylene articulation between 3.3 and 11 years postoperatively because of pain and swelling due to ARMD, except for Case 1 (early revision due to trauma). OA, osteoarthritis; ARMD, adverse reactions to metal debris.

Case	Age and Sex	BMI kg/m^2^	Observed PT after Primary Arthroplasty	Time to Revision after Primary Arthroplasty	Diagnosis	Cup Incli-nation	Cup Ante-version	Symptom	Cup Revised	Stem Revised
Case1 (trauma)	66	33.8		0.33-year	OA	42.1	14.6	Pain, Cup loosening	+	
Case2	73	26.3	2.2-year	3.33-year	OA	53.3	14.0	Pain, Cup loosening	+	
Case3	81	22.5	3.3-year	6-year	OA	46.1	30.2	Pain, Cup loosening	+	
Case4	61	19.9	9.2-year	9.33-year	OA	62.6	11.2	Pain, Cup loosening	+	+
Case5	74	22.7	5.3-year	9.5-year	OA	53.3	9.7	Pain, Cup loosening, Stem osteolysis	+	+
Case6	60	28.1	2.5-year	11-year	OA	58.1	12.0	Pain, Cup loosening	+	
Case7	72	24.6	2.8-year	11-year	OA	73.2	12.6	Pain, Cup osteolysis	+	
Case8	77	25.8	2.4-year	11-year	OA	47.8	17.1	Periprosthetic fracture of femur		+

**Table 2:** Patient demographics and radiological evaluation among hips without pseudotumor (PT), with PT but no revision surgery, and with revision surgery. ns: not significant.

	Hips without PT (21 hips)	Hips with PT but No Revision Surgery (15 hips)	Hips with Revision Surgery (8 hips)	*p*
Female: hip (%)	17 (81.0%)	11 (73.3%)	6 (75.0)	ns
Age (years)	62.2 ± 9.3	64.5 ± 8.3	70.8 ± 7.5	ns
BMI (kg/m^2^)	22.9 ± 2.6	23.3 ± 3.1	25.5 ± 4.2	ns
Head diameter (mm)	45.3 ± 2.6	46.7 ± 2.6	46.3 ± 3.1	ns
Cup inclination angle (°)	43.8 ± 8.5	43.2 ± 9.5	54.6 ± 10	<0.05
Cup anteversion angle (°)	11.4 ± 3.2	11.7 ± 5.0	15.1 ± 6.5	ns
Cup OL/Loosing hip (%)	1 (4.8%)	1 (6.7%)	8 (100%)	<0.0001
Stem OL hip (%)	0 (%)	1 (6.7%)	2 (25%)	*p* = 0.058

**Table 3:** Clinical evaluation in revision cases (eight hips). ns: not significant.

	Evaluation before Revision	Final Evaluation	*p*
JOA	51.1 ± 16.8	72.9 ± 7.7	<0.005
Pain	22.5 ± 10.0	35.6 ± 4.2	<0.005
ROM	12.8 ± 2.8	17.1 ± 1.2	<0.005
Walk	6.9 ± 2.6	9.4 ± 5.6	ns
ADL	9.0 ± 3.9	10.8 ± 4.7	ns

**Table 4:** Clinical evaluation at the final evaluation of hips without pseudotumor (PT), with PT but no revision surgery, and with revision surgery. ns: not significant.

Final Evaluation	Hips without Pseudotumor (PT) (21 hips)	Hips with PT but No Revision Surgery (15 hips)	Hips with Revision Surgery (8 hips)	*p*
JOA	85.1 ± 14.1	85.1 ± 11.2	72.9 ± 7.7	<0.05
Pain	37.1 ± 4.9	37.0 ± 3.2	35.6 ± 4.2	ns
ROM	17.5 ± 2.2	16.6 ± 2.4	17.1 ± 1.2	ns
Walk	15.0 ± 6.0	15.9 ± 5.9	9.4 ± 5.6	<0.05
ADL	15.5 ± 5.1	15.6 ± 3.6	10.8 ± 4.7	<0.05

## Data Availability

Data are contained within the article and Supplementary Materials.

## Supplementary Materials

The following supporting information can be downloaded at: https://www.mdpi.com/article/10.3390/jcm13061525/s1, Figure S1. Changes in pseudotumor (PT) positive frequency over time; Figure S2. MRI images of first observed PT of revised cases 3, 5, 6, 7 and 8; Figure S3. Anteroposterior (AP) radiographs of revised cases before and after revised arthroplasty.
