# Supplementary material for: Long-Term Outcome of Metal-on-Metal Total Hip Arthroplasty with Modular Neck Stem
Source: J Clin Med. 2024 Mar 7;13(6):1525. doi: 10.3390/jcm13061525 (PMC10970930; doi:10.3390/jcm13061525)

Figure S1：Changes in pseudotumor (PT) positive frequency over time

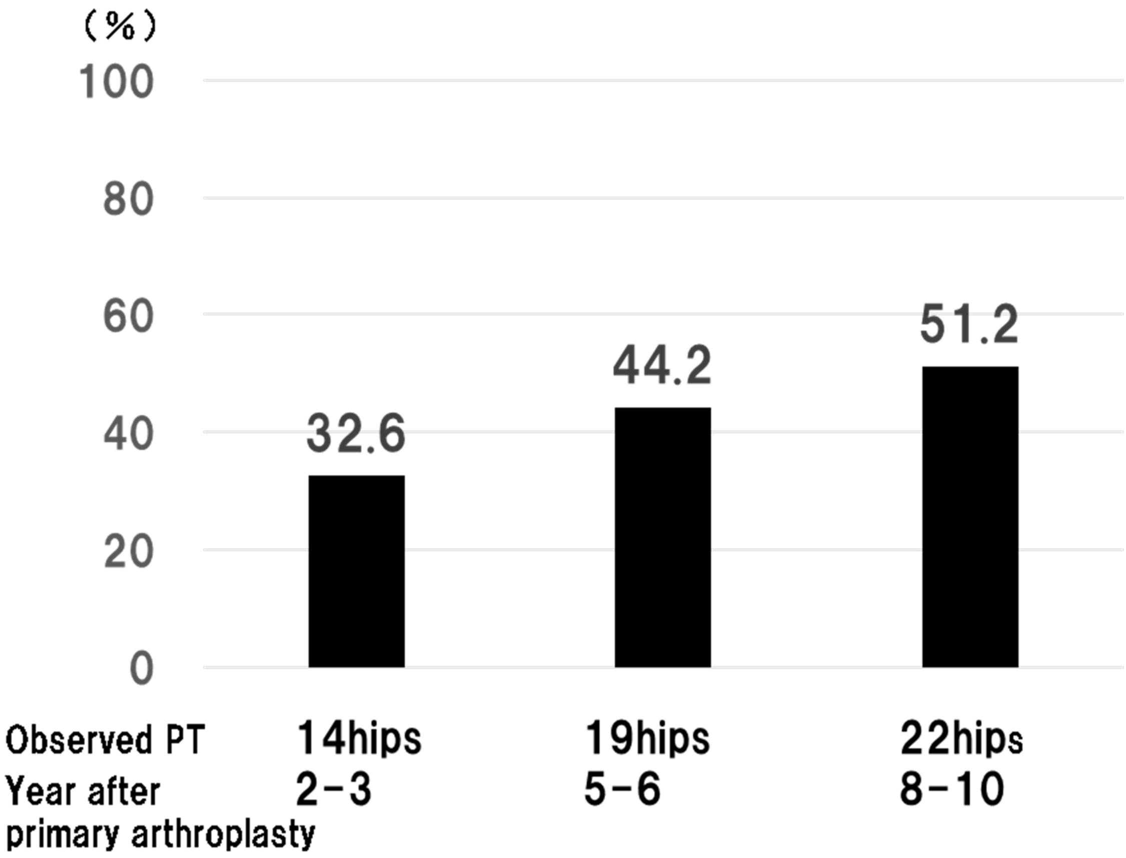

Figure S2：MRI images at first observed PT of revised case3, 5, 6, 7 and 8

Case3 Case5 Case6 Case7 Case8

Axial plane

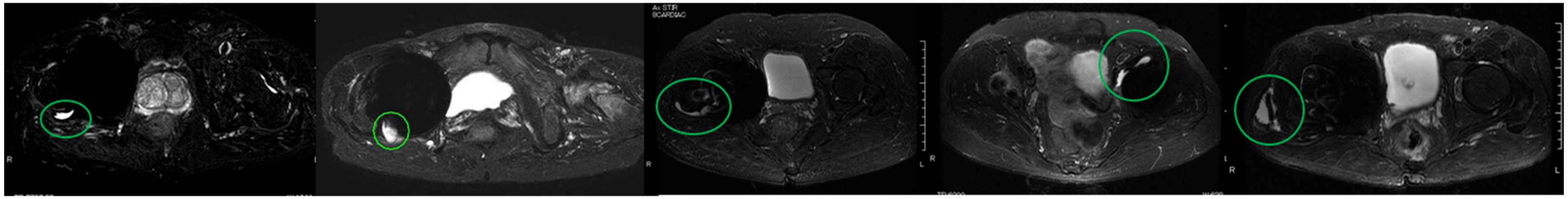

Coronal plane

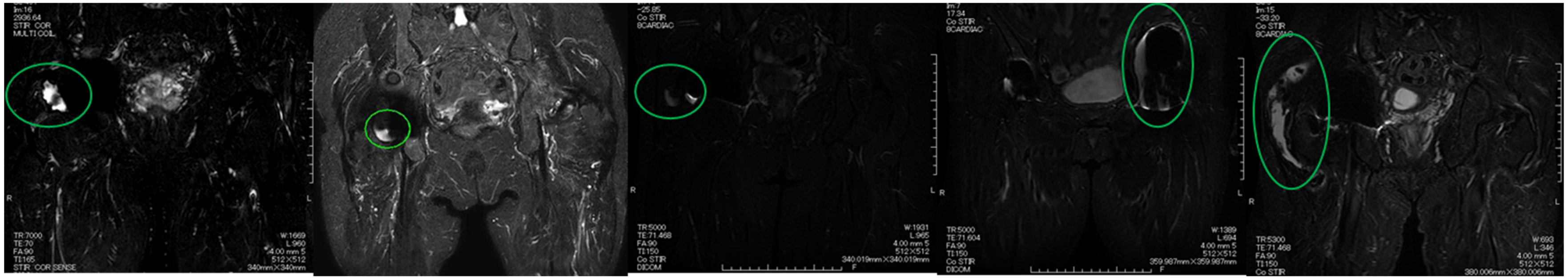

Figure S3：Anteroposterior (AP) radiographs of revised cases before and after revised arthroplasty

Case 1 Case2 Case3 Case4 Case5 Case6 Case7 Case8

Implant failure

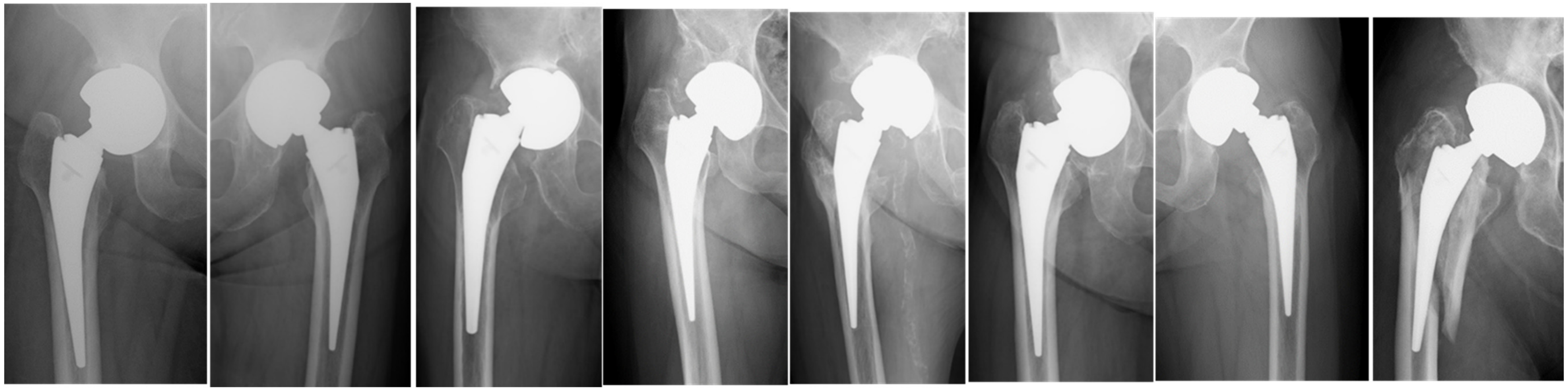

Revised THA

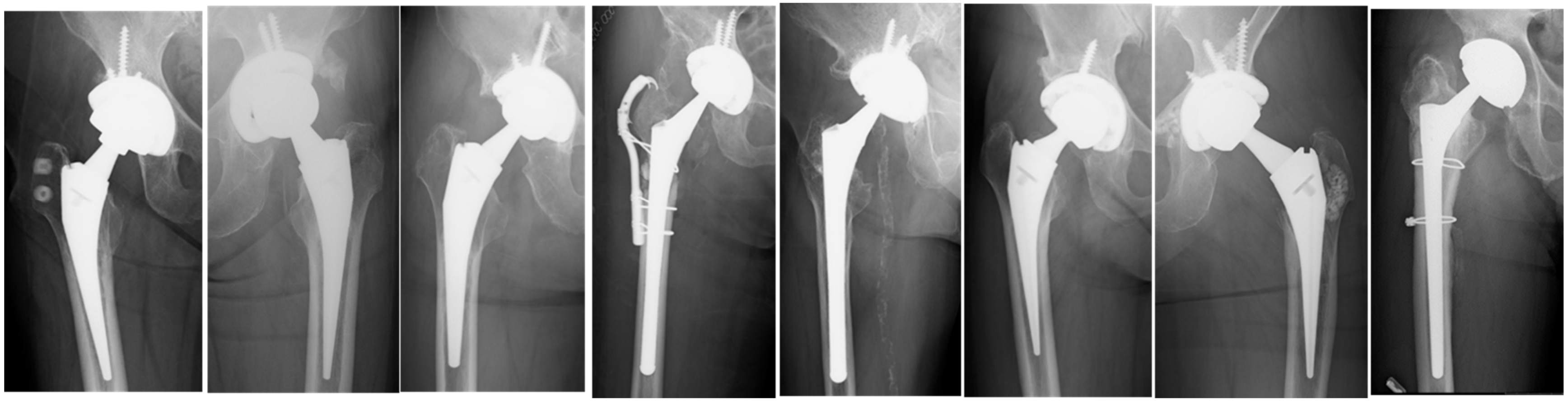

Supplement: Supplementary file 1 [file jcm-13-01525-s001.zip › jcm-2798432-supplementary.pdf]
